# Spatial parasitology and the unmapped human helminthiases

**DOI:** 10.1017/S0031182023000045

**Published:** 2023-04

**Authors:** Catherine G. Schluth, Claire J. Standley, Shweta Bansal, Colin J. Carlson

**Affiliations:** 1 Department of Biology, Georgetown University, Washington, DC, USA; 2 Department of Microbiology and Immunology, Georgetown University Medical Center, Washington, DC, USA; 3 Center for Global Health Science and Security, Georgetown University Medical Center, Washington, DC, USA

**Keywords:** Bayesian modeling, ecological niche modeling, helminth parasites, medical geography, parasite biogeography, spatial statistics, systematic review

## Abstract

Helminthiases are a class of neglected tropical diseases that affect at least 1 billion people worldwide, with a disproportionate impact on resource-poor areas with limited disease surveillance. Geospatial methods can offer valuable insights into the burden of these infections, particularly given that many are subject to strong ecological influences on the environmental, vector-borne or zoonotic stages of their life cycle. In this study, we screened 6829 abstracts and analysed 485 studies that use maps to document, infer or predict transmission patterns for over 200 species of parasitic worms. We found that quantitative mapping methods are increasingly used in medical parasitology, drawing on One Health surveillance data from the community scale to model geographic distributions and burdens up to the regional or global scale. However, we found that the vast majority of the human helminthiases may be entirely unmapped, with research effort focused disproportionately on a half-dozen infections that are targeted by mass drug administration programmes. Entire regions were also surprisingly under-represented in the literature, particularly southern Asia and the Neotropics. We conclude by proposing a shortlist of possible priorities for future research, including several neglected helminthiases with a burden that may be underestimated.

## Graphical abstract

**Figure:**
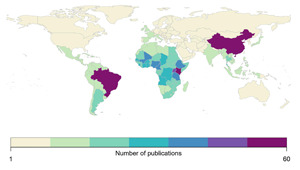

## Introduction

Infections with parasitic worms, or *helminthiases*, have a massive global burden on human health. More than a billion people are infected with soil-transmitted helminths alone, with a total burden of over 3.3 million disability-adjusted life years (DALYs) (Pullan *et al*., 2014); these may be underestimates, given more recent estimates that hookworms alone may account for more than 4 million DALYs (Weatherhead *et al*., 2017). The burden of these infections is highly heterogeneous over space, from case clustering at the community level up to the global scale. Helminthiases persist and recur disproportionately where healthcare systems are too limited for routine treatment, preventative therapies and case management; but these areas are, conversely, often the places where disease surveillance is most limited, and so the burden of these infections is most poorly characterized.

Many previous studies have therefore identified geospatial analysis as a key part of scientific and clinical work on helminthiases. As a basic tool of descriptive epidemiology, maps are one of the simplest and easiest ways to visualize data, communicate risk and engage local communities in participatory research methods. Moreover, geospatial modelling can help fill knowledge gaps about the prevalence or incidence of infectious diseases in under-sampled regions, turning clinical data into a more continuous view of transmission. With enough data, this approach can be used to translate local prevalence surveys into regional and global estimates of incidence or burden. Along the way, geospatial modelling often illuminates environmental and social risk factors for the disease, and perhaps most importantly, helps practitioners target, evaluate and improve interventions.

Previous reviews of infectious disease cartography have evaluated research effort and set priorities for future pathogens to map (Hay *et al*., 2013; Pigott *et al*., 2015), but have only minimally addressed the human helminthiases. A systematic analysis of research trends could help identify the limitations of existing data, target interventions more effectively and broaden the scope of helminth research and control. Here, out of over 6000 candidate mapping studies, we examine a total of 485 scientific studies that developed empirical maps of over 200 helminth species known to infect humans. From these studies, we evaluate trends in research methodology and scope, highlight global gaps in research effort and propose a list of neglected helminthiases that researchers (and surveillance systems) could prioritize in future geospatial studies.

## Methods

### Identifying candidate species

To compile a list of human-associated helminth species, we used a recently published dataset of host–parasite associations curated by the Natural History Museum in London (Dallas, 2016). From these data, we compiled a list of human helminthiases by searching for associations with *Homo sapiens*, and recording the number of references listed for each species. There were 407 helminth species on this initial list. To verify whether each helminth species is still considered taxonomically valid and is capable of infecting humans, we manually searched for records of human infection for each species. In Google Scholar, we used the search queries ‘[species name]’ and ‘human*’ to search for records of human infection. In Google, we used the search queries ‘[species name]’ and ‘syn*’ to determine if species with no records or only old records of human infection have since been renamed. We removed a species from the study if we could find no evidence that the species infects humans, the species name was found to be synonymous with a more recent species name on the list, there was conflicting evidence as to whether the species can infect humans or the species was found to infect humans only as a hybrid with another helminth species. This left a total of 232 taxonomically valid human helminthiases.

### Systematic review

Taking an iterative approach to data collection, we used *Wuchereria bancrofti* as a test species to determine which search terms would be most effective to retrieve helminth mapping studies. On Google Scholar, we used the search queries ‘*Wuchereria bancrofti*’ and ‘mapping’. Based on an informal analysis of the results, we selected a final set of keywords that consistently signalled that empirical spatial analysis was undertaken. Our final search query was ‘[species name]’ and (‘SaTScan’ or ‘MaxEnt’ or ‘spatial cluster*’ or ‘spatial analysis’ or ‘geospatial’ or ‘ecological niche model*’ or ‘mapping’ or ‘nearest neighbor’ or ‘spatial GLM*’). To identify candidate studies, we searched PMC and PubMed for these terms with each of the 232 helminth species, one by one. Our search may have missed mapping studies that were written and published in other languages, as well as grey literature produced by health ministries or non-governmental organizations; as such, our study represents only a snapshot of the retrievable literature.

Our literature review was conducted between November 2018 and May 2019 (and is, as such, limited to the pre-pandemic period), following PRISMA guidelines (see Fig. S1). We included studies in our dataset if they presented novel data or a new modelling product representing the known or predicted spatial distribution of a helminth species, the condition(s) it causes, and/or the medication used to treat it; studies were excluded if they did not use empirical data to generate either a map-based visualization or a spatial model of human helminthiasis infection over space. Two authors conducted the review and both verified each study that was selected for inclusion. In total, we found 485 studies that mapped a total of 45 helminth species. For each study in the final dataset and analysis, we recorded all available information on: the full binomial nomenclature of helminth species being mapped; the citation for and link to each study; the year each study was conducted; the spatial scope of each study; the specific methodologies used in each mapping effort; the sample size and type in each study; whether each study addressed uncertainty and population at risk; whether each study examined coendemicity and/or coinfection among helminth species or between helminth species and other diseases and whether each study publicly archived the associated data.

### Ontology of study methodology

To develop an ontology of research methods (Augustijn-Beckers *et al*., 2022), we examined the mapping methodology of studies and grouped them into a handful of non-exclusive categories, adapted from a previously published study on tick-borne disease mapping (Lippi *et al*., 2021):

- **Grey data** describe the presentation of spatial point data of either cases or positive–negative testing results. For attempts to develop *post hoc* databases and risk maps, these are data that could be heads-up digitized and reused (provided they have not been jittered for data security and anonymization).
- **Prevalence mapping** refers to raw or aggregated prevalence data presented on a map; like grey data, this is a presentation of raw data, but with more granularity with respect to intensity of transmission. For our purposes, this also includes non-prevalence quantitative measures of transmission intensity, e.g. fecal egg counts.
- **Prevalence modelling** refers to using statistical models to analyse or reconstruct patterns of prevalence, involving either a model with an explicit spatial component or spatial covariates (e.g. climate data), or spatial autocorrelation analyses such as Moran's I or autocorrelograms. (E.g. a binomial logistic regression that is stratified by age and sex alone would not qualify for this; such a model incorporating distance to rivers, gridded rainfall or a conditionally autoregressive term would qualify.)
- **Cluster analysis** refers to spatial clustering methods such as SaTSCAN or directional distribution models (standard deviation ellipse), a subset of prevalence modelling focused on identifying specific, discrete points and areas of case clusters or transmission hot spots.
- **Risk mapping** refers broadly to the projection of modelled risk surfaces over a continuous area or at regional levels; this involves at least some amount of inference of risk, from a model of prevalence or occurrence, and visual presentation of modelled results (i.e. not hand-drawn or expert-informed maps).
- **Ecological niche modelling** is a subtype of risk mapping, for when authors explicitly refer to habitat suitability models, ecological niche models or species distribution models as the methodology being used. Risk maps with ecological covariates are arguably habitat suitability models *sensu lato*, but not necessarily part of that specific body of literature.
- Finally, **endemicity mapping** refers to the delineation of known or suspected zones of endemicity or transmission, based on expert knowledge and/or historical or published data reused or consolidated to identify likely zones. For example, this can be the identification of possibly at-risk communities, or the mapping of survey results at the national level for a whole continent.

## Results

### Helminthiases are a topic of growing interest in medical geography

**Fig. 1.**
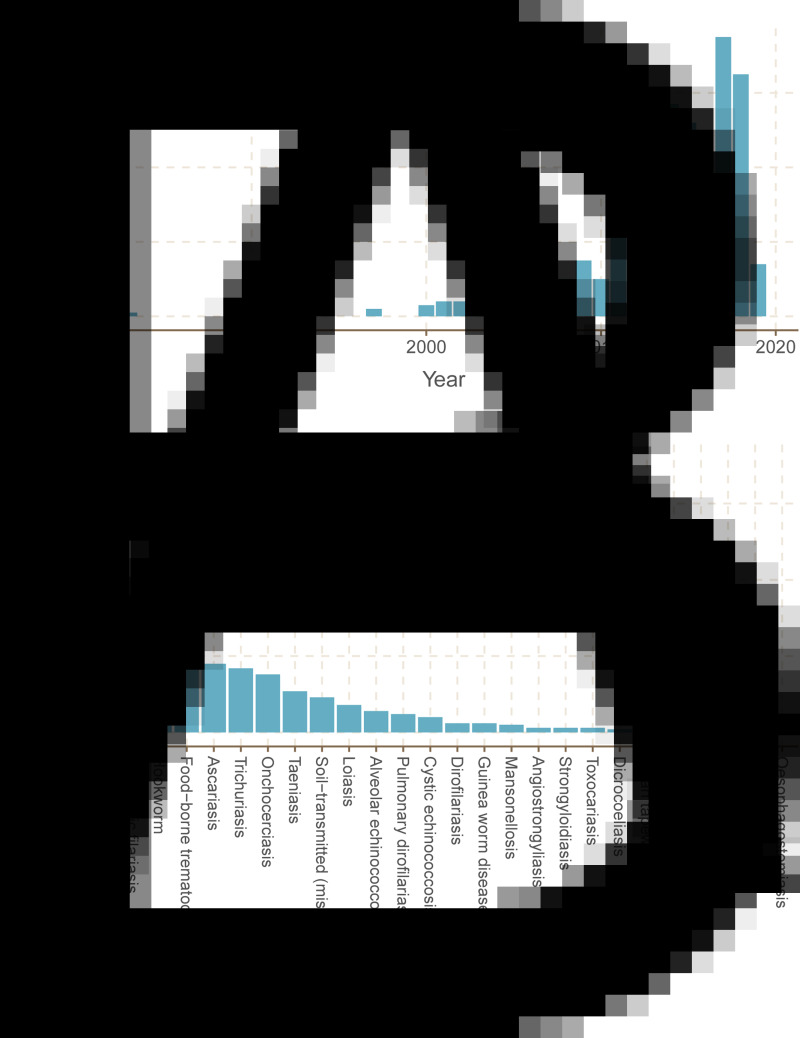
(A) Efforts to map the human helminthiases have increased over time. (B) Spatial data for a few helminthiases make up the majority of all human helminth spatial data. The 45 helminth species with spatial data were grouped together by the conditions they cause (e.g. *Wuchereria bancrofti* and *Brugia malayi* are grouped as lymphatic filariasis).

**Fig. 2.**
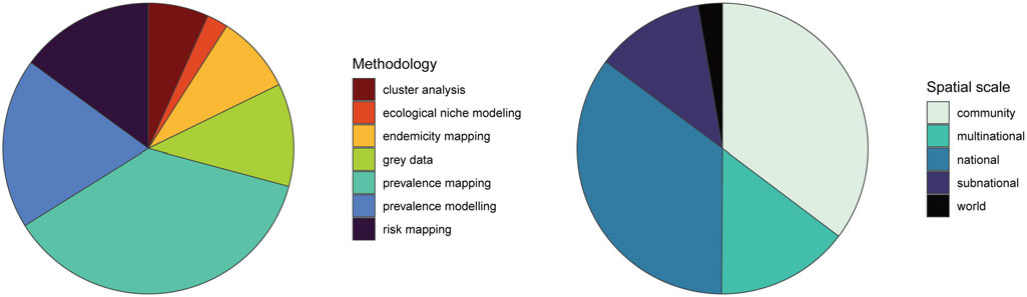
Existing human helminth spatial data predominantly comes from small-scale prevalence mapping studies. Studies containing spatial data on human helminthiases were characterized by spatial scale and methodology, with several studies employing more than 1 methodology.

We found a total of 485 studies that mapped human helminthiases across a mix of scales, regions, pathogens and purposes. The number of helminth mapping studies has steadily increased since 2000 (Fig. 1), and the field is still growing rapidly. Across all time periods, we found that most studies use maps first and foremost as a data visualization tool (case occurrence data or prevalence maps; Fig. 2); however, the last decade has seen a particular shift towards advanced statistical modelling and machine-learning approaches. In particular, tools from ecological niche modelling began to be used around 2007–2010, when the most popular algorithms (MaxEnt and GARP) began to cross over into medical geography. This particular approach to predictive modelling is continuing to become more popular as more disease ecologists become involved in neglected tropical disease research. In the last decade, we also observed a methodological shift away from studies using licensed software such as ArcGIS, and increasingly taking advantage of open-source software such as QGIS and GRASS or console programs such as R and Python. These accessible software can be easily used by researchers and stakeholders without the financial barriers of proprietary software that are prohibitive even for many in the Global North.

### Spatial scale and global gaps

**Fig. 3.**
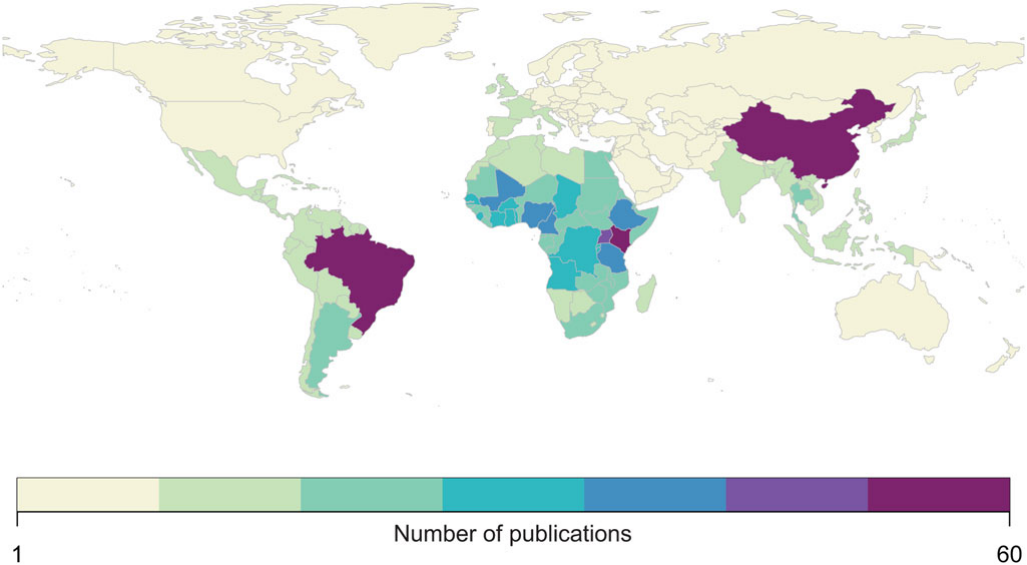
Most published spatial research on human helminthiases describes incidence and burden in sub-Saharan Africa (particularly Kenya and Uganda), China and Brazil.

We found that the vast majority of mapping studies were boots-on-the-ground epidemiological research conducted in a single community or a handful of communities within a single country (Fig. 2). We found relatively few large-scale (multinational to global) prevalence or risk maps, likely because the raw prevalence data are unsynthesized in a modellable format or, for many infections, have never been collected at sufficient scale. We also found that the distribution of research effort has been strikingly uneven (Fig. 3). Hot spots of research in China, Brazil and tropical sub-Saharan Africa reflect a mix of population size, infectious disease burden and unique aspects of the medical parasitology community of practice (e.g. Fiocruz in Brazil). However, we found major research gaps in South and Southeast Asia, the Middle East and Latin America and the Caribbean, despite the high parasite burden faced by many communities in these regions (e.g. Salam and Azam, 2017). Overall, these findings suggest that the global burden of many helminthiases might be underestimated, especially if parasite prevalence is high in research and surveillance cold spots.

### Most human helminthiases are unmapped or undermapped

Of the 232 species targeted by our systematic review, only 45 had any associated studies – indicating nearly 200 unmapped species of human parasites. Out of those 45, a half-dozen of the best-known conditions – the major vector-borne helminthiases (schistosomiasis, lymphatic filariasis and onchocerciasis) and soil-transmitted helminthiases (hookworms, *Ascaris* and *Trichuris*) – account for the vast majority of research effort (Fig. 1). Unsurprisingly, these infections account for the majority of the total burden of helminthiases on global health and poverty. Ascariasis is the most common helminth infection in the world, thought to infect between 737 million and 872 million people worldwide (Vos *et al*., 2017), and is a major cause of stunting and malnutrition in children. *Trichuris* and hookworms have a similarly massive burden, infecting roughly 435 million and 450 million people worldwide, respectively (Vos *et al*., 2017). Schistosomiasis infects between 179 million and 200 million people worldwide (Vos *et al*., 2017), with a high burden especially in HIV-coendemic areas. Aside from these infections, all other human helminthiases are generally presumed to infect fewer than 100 million people worldwide.

### Feedbacks between mapping and interventions

Research effort also reflects feedbacks among mapping work, ease of treatment and scale of interventions. A small number of infections are targeted by mass drug administration (MDA) programmes, both because of cheap widely available treatments and because they account for the highest global burden. These programmes are naturally complimentary with spatial analysis: defining the boundaries of a community, testing people or animals for helminthiases and updating endemicity maps are among the easiest ways to visualize burden and decide on the frequency and distribution of drug administration. This ongoing feedback of prevalence studies, GIS work and targeted drug administration has been a key part of successful MDA efforts over the past 20 years, not just to tailor efforts but also to measure their success and justify ongoing funding. These programmes are therefore the main reason that broad, synthetic data and cartography are possible for a small subset of the best-mapped helminthiases (i.e. soil-transmitted helminths, schistosomiasis, lymphatic filariasis and onchocerciasis). Conversely, we found that most infections without readily available anthelminthic treatments were relatively understudied, or never appeared in our dataset.

**Fig. 4.**
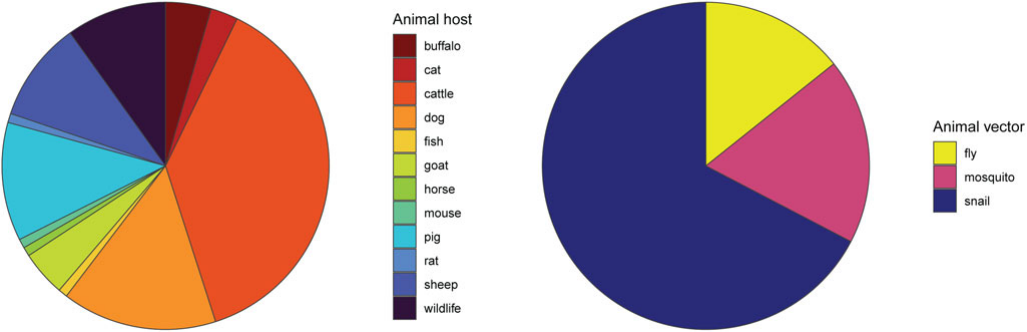
Among studies that map other helminth hosts or helminth vectors, studies mapping less mobile hosts and vectors predominate. Baboons, antelopes and wild boars were classified as wildlife hosts; some studies mapped multiple non-human hosts.

Soil-transmitted helminths are also exceptional in that most have a relatively simple life cycle; as such, prevalence data in humans are usually likely to capture the extent of transmission (though see Nejsum *et al*., 2012). In contrast, many under-represented helminth species were zoonotic, likely because complex life cycles or wildlife hosts make them more challenging targets for surveillance. When non-human hosts are studied, they are almost always livestock (especially cattle and sheep), pets (cats and dogs) or synanthropic wildlife (rats and mice); true wildlife hosts account for a small fraction of studies (Fig. 4). Some of the most understudied helminthiases are the ones that complete parts of their life cycle in hosts that are particularly difficult to sample, such as fish and marine mammals (e.g. *Anisakis* or *Diphyllobothrium*). Similarly, vector-borne transmission adds another layer of ecological influence, which can make ecological models more useful, but primary data collection more challenging; we found more studies collected spatial data on snails (the ‘vectors’, or more accurately intermediate hosts, of schistosomiasis) than on more mobile vectors such as mosquitoes or flies. Surprisingly, even for soil-transmitted species, we found that environmental sampling is nearly never reported (Fig. S2).

### Coinfections, coendemicity and syndemic interactions

We found that a surprising number of mapping studies directly addressed helminth coinfections and coendemicity. Many surveillance programmes – especially those guiding MDA – inherently collect data on multiple helminthiases at once (e.g. Kato-Katz screening can detect *Ascaris*, *Trichuris*, schistosomiasis, hookworms and other eggs of other parasites as well). Studies that map hot spots of coinfection (e.g. Raso *et al*., 2006; Pullan *et al*., 2008; Brooker and Clements, 2009) can help prioritize where treatment might have the greatest social and economic benefits. These approaches can also address more complicated syndemic interactions (Singer *et al*., 2017): for example, onchocerciasis control programmes often use ivermectin, a drug that can cause severe neurological complications or even death when administered to a patient with loiasis (Wanji *et al*., 2018). Increasingly, mapping studies have been used to address *Onchocerca*–*Loa* coendemicity in west and central Africa, helping practitioners to delineate where ivermectin can be administered safely, and where other interventions such as vector control might be safer and more effective.

Finally, we found over a dozen studies that addressed coinfections or coendemicity of helminthiases with other infections. Most of these studies focused on malaria: 3 studies mapped coinfections with hookworms (Brooker and Clements, 2009; Brooker *et al*., 2012; Adu-Gyasi *et al*., 2018), 2 with schistosomiasis (Kabatereine *et al*., 2011; Doumbo *et al*., 2014) and another with lymphatic filariasis (Stensgaard *et al*., 2011). Integrated mapping can address different aspects of syndemic interactions: for example, some helminthiases share a preventable transmission route with other pathogens (e.g. *Anopheles* mosquitoes transmit both malaria and lymphatic filariasis); others are treatable with the same drugs [artemisin, a widely used antimalarial, also targets immature schistosomes (Bergquist and Elmorshedy, 2018); recent evidence suggests ivermectin in blood meals may reduce *Anopheles* mosquito lifespan (Derua *et al*., 2015; Mekuriaw *et al*., 2019)]. Perhaps the most elusive facet of helminthiases' burden is their immunomodulatory effects, which can have unpredictable impacts on other diseases: for example, while *Schistosoma mansoni* or hookworm infections may increase susceptibility to malaria, *Schistosoma haematobium* infections may confer protection against severe malaria (Adegnika and Kremsner, 2012; Donohue *et al*., 2019). In any of these contexts, helminthiases are worth considering in broader efforts to measure and reduce the global burden of the disease.

## Discussion

Here, we screened over 6000 studies, and found extensive literature on the human helminthiases that incorporated geospatial approaches (nearly 500 studies). However, we found that most of these studies were focused on a half-dozen or so parasites with a simple life cycle, available low-cost treatments and the greatest global burden – the circumstances that make elimination programmes a cost-effective investment. For the vast majority of human helminthiases, we found no geospatial data or analysis of any kind in the studies we reviewed. Some of these parasites may only sporadically infect humans, but several others are known to have an uncertain but likely medium-to-high global burden. Often, these neglected helminthiases have a complex (zoonotic or vector-borne) life cycle that both complicates surveillance and limits the feasibility of vertical control programmes (especially if elimination is precluded by non-human reservoirs). For these neglected infections, there are many opportunities for mapping work to both establish a clearer baseline on global burden, and to support One Health interventions that include vector control, community sanitation, food safety, livestock vaccination, routine deworming of household pets and similar practices. In service of this goal, we propose a shortlist of several notable but neglected human helminthiases that were underrepresented in the literature (Table 1).

Table 1. An (incomplete) list of high-priority helminthiases for geospatial research

1. Angiostrongyliasis (*Angiostrongylus cantonensis*)
2. Hepatic and intestinal capillariasis (*Capillaria hepatica*, *Capillaria philippinensis*)
3. Carcinogenic food-borne trematodiases (*Clonorchis sinensis*, *Opisthorchis viverrini*)
4. Guinea worm disease (*Dracunculus medinensis*)
5. Echinococcosis (*Echinococcus granulosus*, *Echinococcus multilocularis*)
6. Gastrodiscoidiasis (*Gastrodiscoides hominis*)
7. Dwarf tapeworm (*Hymenolepis nana*)
8. Mansonellosis (*Mansonella perstans*, *Mansonella ozzardi*, *Mansonella streptocerca*)
9. Strongyloidiasis (*Strongyloides stercoralis*)
10. Taeniasis and cysticercosis (*Taenia solium*)

### Opportunities for global burden (re-)estimation

Several helminthiases with a global distribution have a likely high but uncertain burden, which could be clarified by a coordinated data synthesis and geospatial modelling effort – potentially motivating more global investment in prevention and treatment. For example, see the sub-sections below.

#### Echinococcosis

*Echinococcus granulosus* and *Echinococcus multilocularis* are zoonotic tapeworms that cause cystic and alveolar echinococcosis, respectively. These infections typically remain asymptomatic for years until cysts grow large enough to disrupt organ function; when they rupture, or (in the latter case) result in liver failure, case fatality rates are relatively high (Wen *et al*., 2019). Between the 2 infections, recent estimates place the global burden roughly 19 000 deaths per year out of nearly a million active cases (World Health Organization, 2016). Treatment is difficult, and may require surgery, but infections can also be prevented with a One Health strategy that includes slaughterhouse hygiene and deworming dogs. Global summaries of prevalence data at the national or subnational level have recently been compiled not just for human hosts, but also wildlife and domesticated hosts (Deplazes *et al*., 2017); these data could be readily applied to more detailed, fine-scale geospatial modelling.

#### Taeniasis and cysticercosis

A zoonotic parasite of swine, *Taenia solium* is endemic worldwide in communities with poor sanitation and consumption of undercooked pork. Intestinal infection with the adult tapeworm is usually mild, but fecal–oral transmission between humans leads to paratenic infections that can form on the brain or on the spinal cord. These severe infections cause at least 28 000 deaths annually (World Health Organization, 2016), and account for at least a third of all epilepsy cases in endemic areas (Gripper and Welburn, 2017). Estimates range between 2 and 6 million infections worldwide, but some upper-end regional estimates (e.g. 1.2 million attributable epilepsy cases in India alone; Rajshekhar, 2016) suggest these may be global underestimates. A high-resolution global estimate of burden could help target One Health interventions pairing MDA for taeniasis with pig vaccination, which can eliminate the pathogen over just 4–5 years (Braae *et al*., 2016).

#### Strongyloidiasis

*Strongyloides stercoralis* is a soil-transmitted nematode that infects tens of millions of people in rural communities without proper sanitation. Strongyloidiasis is often asymptomatic, but can be life-threatening in immunocompromised individuals. Our systematic review identified just 3 efforts to map this parasite – all national or community studies – highlighting an opportunity to consolidate existing surveillance data, and develop high-resolution maps of endemicity and burden. One recent study takes an important step towards filling this gap by developing a global ecological niche model for strongyloidiasis (Fleitas *et al*., 2022), but data remain limited and more systematic efforts are needed.

#### Hymenolepiasis

The dwarf tapeworm *Hymenolepis nana* is one of the most common cestode parasites of humans. These infections are generally asymptomatic in adults, but more severe in children, especially when comorbid with malnutrition (Cabada *et al*., 2016). Estimates of regional *H. nana* prevalence vary substantially, ranging from 0.2 to 28.4% in Asia and from 0.9 to 23% in the Americas (Vilchez Barreto *et al*., 2017). Our literature review found only 2 mapping studies on *H. nana*, both community-based studies in Angola and Ghana (Soares Magalhães *et al*., 2013; Adu-Gyasi *et al*., 2018). Future work could consolidate fine-scale surveillance datasets, and align them with other geospatial research on malnutrition and stunting (e.g. Osgood-Zimmerman *et al*., 2018).

### Opportunities for global distribution or risk mapping

For several other helminthiases with a global distribution, a baseline global map of endemicity – or a risk map of the zoonotic niche and the socioenvironmental risk factors for infection – might be a substantial step forward. For example, see the sub-sections below.

#### Angiostrongyliasis

More commonly called rat lungworm, *Angiostrongylus cantonensis* is a rare but emerging infection endemic to Asia and the Pacific that causes eosinophilic meningitis (Cowie, 2013). Some infections are self-limiting, but others may cause significant neurological damage or death. The geographic range of the parasite has expanded over time, potentially facilitated by climate change and human movement, with several thousand cases reported worldwide (Cowie, 2013; Martins *et al*., 2015). Existing literature on the parasite's global distribution has worked from sparse data (Martins *et al*., 2015; Lu *et al*., 2018), and future work could more directly address the parasite's zoonotic niche in both intermediate hosts (snails and slugs) and the ultimate host (rats).

#### Mansonellosis

An infection that has been called ‘the most neglected human filariasis’ (Mediannikov and Ranque, 2018), *Mansonella perstans* alone is thought to infect over 100 million people (Ta-Tang *et al*., 2018), yet there are currently no large-scale control programmes targeting any *Mansonella* species. Our literature review identified 9 studies mapping mansonellosis, including a map of global endemicity. Future research could aim to generate a high-resolution global risk map to guide vector control efforts, particularly given that its *Culicoides* midge vectors also transmit several emerging infections (including bluetongue virus, Oropouche virus and African horse sickness).

#### Guinea worm disease

Unlike other infections on our shortlist, guinea worm disease cannot be considered neglected. Decades of control efforts brought *Dracunculus medinensis* close to being the first globally eradicated parasite, though canine reservoirs now jeopardize that progress (Molyneux and Sankara, 2017; Wilson-Aggarwal *et al*., 2021). Despite the small number of remaining transmission foci, guinea worm was once found throughout the tropics. Little geospatial data have been consolidated on this century-long range contraction; the best available maps of its original range are hand-drawn estimates from the 1950s (May, 1952). Remapping the historical distribution of *D. medinensis* with modern technology and modelling methods could offer some insights into the previous successes of eradication campaigns, clarify the ongoing role of zoonotic reservoirs (Boyce *et al*., 2020) and offer fresh motivation to eliminate guinea worm disease on the last remaining continent.

### Opportunities for regional and community-based mapping

For a handful of the rarest or most neglected helminthiases, more boots-on-the-ground studies of parasite prevalence in local communities are still needed, forming the quantitative basis of broader estimates of parasite distribution and prevalence.

#### Carcinogenic food-borne trematodiases

*Opisthorchis viverrini* and *Clonorchis sinensis* are trematode parasites of fish (Sripa *et al*., 2011), both of which seriously increase the risk of bile duct cancer (cholangiocarcinoma). The burden of *O. viverrini* is localized to southeast Asia and particularly to Thailand, which has the highest incidence of cholangiocarcinoma worldwide (Banales *et al*., 2016); opisthorchiasis prevalence attains 70% in some communities, costing the country an estimated 120 million USDannually (Sripa *et al*., 2011). *Clonorchis sinensis* has a much wider range throughout east Asia, infecting an estimated 35 million people (Kim *et al*., 2016). Our systematic review found no community-based mapping efforts for *C. sinensis*, and very few for *O. viverrini*.

#### Capillariasis

*Capillaria hepatica* and *Capillaria philippinensis* cause hepatic and intestinal capillariasis, respectively. The former is believed to only infect humans rarely, while outbreaks of the latter have been sizable; both can be deadly, and are challenging to diagnose (Li *et al*., 2010). Human infections with both parasites are apparently rare but widespread: *C. philippinensis* can be found throughout Asia and parts of North Africa and the Middle East, and *C. hepatica* is found in wildlife worldwide (Saichua *et al*., 2008; Li *et al*., 2010). However, our literature search did not identify any efforts to map either species.

#### Gastrodiscoidiasis

*Gastrodiscoides hominis* is a poorly characterized food-borne zoonotic fluke (Chai *et al*., 2009), capable of causing diarrhoea, malnutrition and death. *Gastrodiscoides hominis* is thought to be highly prevalent in India especially, but cases occur throughout Asia and Africa (Dada-Adegbola *et al*., 2004; Mas-Coma *et al*., 2005). Our literature search did not identify any efforts to map *G. hominis* infection.

### Broader opportunities for geospatial research

In less than a century, geospatial research on the human helminthiases has advanced from hand-drawn world maps (May, 1952) to fine-scale maps of transmission, comorbidity and intervention priorities. This advance has been driven as much by the advent of powerful statistical methods such as Bayesian spatiotemporal models (Stensgaard *et al*., 2011; Chammartin *et al*., 2013; Scholte *et al*., 2014) as it has been by the growing availability of national and transnational datasets of sufficient scale to power those models. Because these datasets only exist for a handful of infections, the benefits of these methodological advances have yet to be felt in many areas of medical parasitology. In this study, we find that there are significant opportunities to expand research along these lines, and thereby improve scientific and clinical understanding of some of the most neglected tropical and temperate diseases.

We conclude by noting that infectious diseases are rarely a stationary target for mapping and modelling. While geospatial methods are already widely used to track the progress of elimination programmes, we finally note that these tools will also have value as climate change drives shifts in the geographic distribution and burden of helminthiases, their vectors and their wildlife reservoirs. As temperature rises, the range of hookworms is expected to expand towards the southernmost regions of Africa (Blum and Hotez, 2018); as *Anopheles* mosquitoes and other vectors undergo geographic range shifts (Carlson *et al*., 2019), they may introduce parasites into new regions or undo progress towards elimination. In other cases, climate change may make environmental conditions less hospitable for parasites (Carlson *et al*., 2017): angiostrongyliasis may lose range as China warns (York *et al*., 2014), and the snail intermediate hosts of schistosomiasis may begin to shrink as their habitats grow hotter and more arid (Blum and Hotez, 2018). Ecological modelling will help triage the greatest climate-related risks to human health, while other mapping methods will be critical to document the impacts of climate change in real time. In that light, One Health data on parasitic infections in humans, livestock, domestic animals and wildlife all become even more valuable – particularly when shared openly with sufficient geospatial metadata for reuse.

## Data Availability

All data will be made available on Zenodo at the time of publication.

## Supplementary material

For supplementary material accompanying this paper visit https://doi.org/10.1017/S0031182023000045. click here to view supplementary material
