# Supplementary material for: Spatial parasitology and the unmapped human helminthiases
Source: Parasitology. 2023 Jan 12;150(4):391–9. doi: 10.1017/S0031182023000045 (PMC10090474; doi:10.1017/S0031182023000045)
Supplement: Supplementary file 1 [file S0031182023000045sup.zip › S0031182023000045sup002.docx]

**Figure S1.** Systematic review procedure, following PRISMA reporting guidelines.

**Figure S2.** Diagnostic methods used to collect primary data on parasitic infections in 357 studies that report new data from field surveillance.


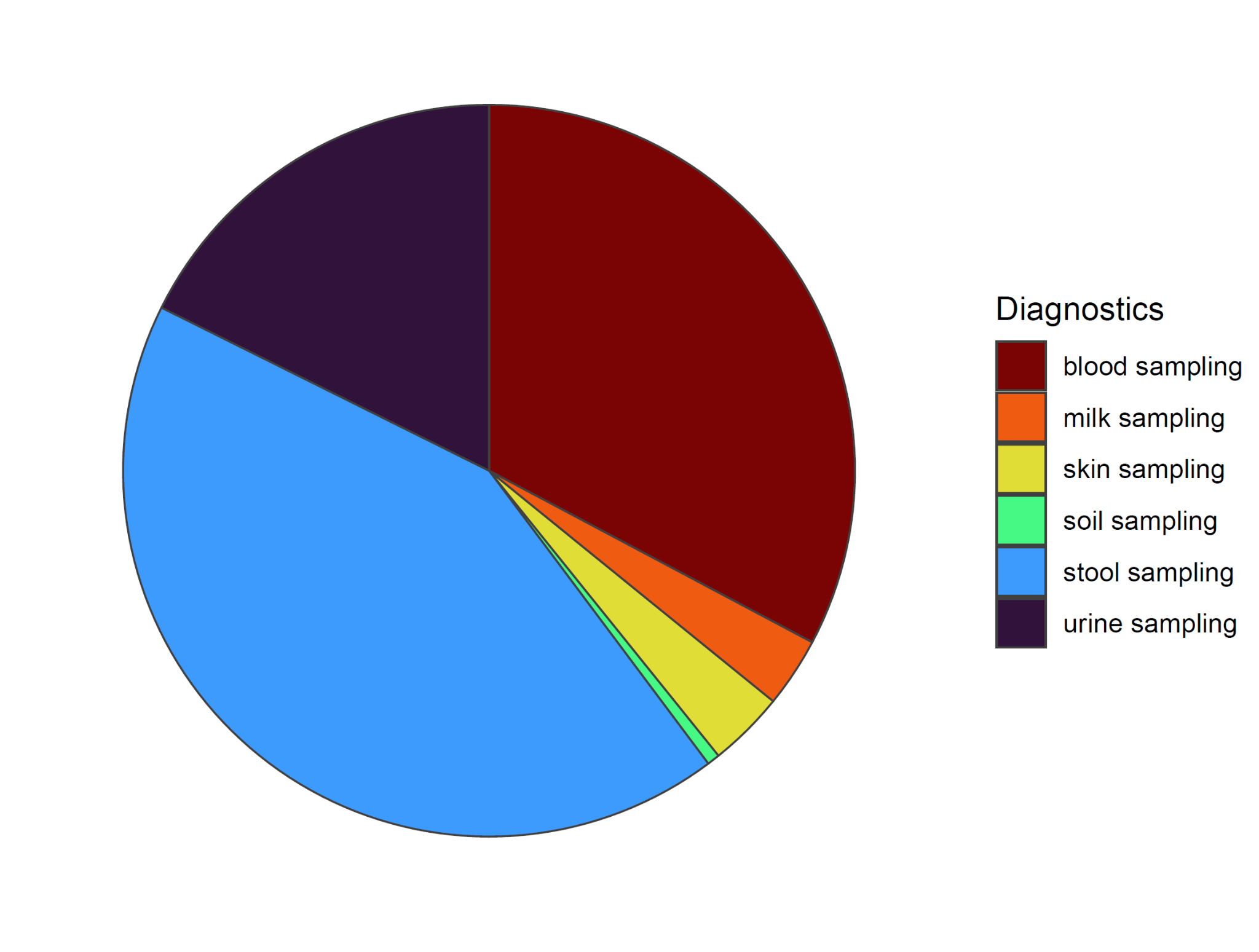


**Table S1.** Studies that report disease names but not Latin names have been removed from this table, as have those identifying parasites only to genera, but those studies are shown in Figure 1.

| **Latin binomial name** | **Number of references** |
| --- | --- |
| *Schistosoma mansoni* | 89 |
| *Wuchereria bancrofti* | 76 |
| *Schistosoma haematobium* | 67 |
| *Ascaris lumbricoides* | 45 |
| *Trichuris trichiura* | 42 |
| *Schistosoma japonicum* | 38 |
| *Onchocerca volvulus* | 37 |
| *Fasciola hepatica* | 36 |
| *Loa loa* | 18 |
| *Taenia solium* | 17 |
| *Echinococcus mutilocularis* | 14 |
| *Dirofilaria immitis* | 12 |
| *Echinococcus granulosus* | 10 |
| *Opisthorchis viverrini* | 9 |
| *Taenia saginata* | 8 |
| *Dirofilaria repens* | 6 |
| *Necator americanus* | 6 |
| *Dracunculus medinensis* | 5 |
| *Fasciola gigantica* | 5 |
| *Brugia malayi* | 4 |
| *Mansonella perstans* | 4 |
| *Angiostrongylus cantonensis* | 3 |
| *Clonorchis sinensis* | 3 |

**Table S1,** continued.

| *Schistosoma guineensis* | 3 |
| --- | --- |
| *Strongyloides stercoralis* | 3 |
| *Toxocara cati* | 3 |
| *Dicrocoelium dendriticum* | 2 |
| *Hymenolepis nana* | 2 |
| *Mansonella ozzardi* | 2 |
| *Opisthorchis felineus* | 2 |
| *Schistosoma intercalatum* | 2 |
| *Schistosoma mekongi* | 2 |
| *Taenia asiatica* | 2 |
| *Thelazia callipaeda* | 2 |
| *Trichinella britovi* | 2 |
| *Anisakis pegreffi* | 1 |
| *Anisakis physeteris* | 1 |
| *Echinostoma revolutum* | 1 |
| *Fasciolopsis buski* | 1 |
| *Hymenolepis diminuta* | 1 |
| *Mansonella streptocerca* | 1 |
| *Oesophagostomum bifurcum* | 1 |
| *Paragonimus skrjabini miyazakii* | 1 |
| *Paragonimus westermani* | 1 |
| *Schistosoma malayensis* | 1 |
